# Complete Genome Sequence of Limosilactobacillus reuteri Strain VHProbi M07, Isolated from Breast Milk

**DOI:** 10.1128/mra.00764-22

**Published:** 2022-10-26

**Authors:** Hongchang Cui, Songjie Wu, Zhi Duan

**Affiliations:** a Qingdao Vland Biotech Group Co., Ltd., Qingdao, China; University of Maryland School of Medicine

## Abstract

The probiotic strain of Limosilactobacillus reuteri VHProbi M07 was isolated from fresh breast milk. Here, we report the whole-genome sequence of this strain. The whole genome comprised 2,041,530 bp with a GC content of 38.89%.

## ANNOUNCEMENT

A total of 1 mL of fresh breast milk was serially diluted, and 10^−9^ dilutions were spread onto the de Man-Rogosa-Sharpe agar medium (1). The inoculum was incubated at 37°C for 48 h under anaerobic conditions. This research was carried out in accordance with the Declaration of Helsinki. A strain designed VHProbi M07 was identified by 16S rRNA sequencing according to the method described by Liu (2). Results showed that the strain VHProbi M07 belonged to the species Limosilactobacillus reuteri. A combined strategy of the Illumina HiSeq 2500 platform and the Pacific Bioscience RSII platform was used to sequence the whole genome of this strain. The strain was cultured for DNA extraction using the same culture condition as that for strain enrichment. Total DNA was extracted using the genomic DNA purification kit (Promega, USA) following its instructions for the Illumina and the PacBio sequencing. The Illumina paired-end sequencing library was generated using the NEXTflex rapid DNA sequencing (DNA-seq) kit (Bio Scientific, USA). The prepared libraries were then used for Illumina sequencing on the Illumina HiSeq 2500 machine. For PacBio sequencing, an aliquot of 15 μg DNA was sheared into ~10-kb fragments and then the fragments were purified, end paired, and ligated with the SMRTbell adapters following the manufacturer’s recommendations (Pacific Biosciences, CA). The resulting sequencing library was purified three times using 0.45× volumes of Agencourt AMPure XP beads (Beckman Coulter Genomics, MA) following the manufacturer’s recommendations. Finally, 7,097,864 raw reads and 7,039,750 clean reads were generated using the Illumina sequencing platform. A total of 198, 94 reads (*N*_50_, 252,381 bp) were generated using the PacBio RS II sequencing platform. The complete genome sequence was assembled using both the Illumina reads and the PacBio reads. The Illumina-generated reads were quality filtered by Sickle v1.33 (https://github.com/najoshi/sickle), and low-quality reads were removed before assembly. SOAPdenovo v2.04 was used to generate scaffolds using the Illumina reads (3). The PacBio reads were quality controlled using P_Fetch and P_Filter modules of SMRT analysis v2.3 (4). The PacBio reads were assembled into a contig using Unicycler v0.4.8 (5). Pilon v1.22 was used for the performance of the error check of the PacBio assembly results (6). The last circular step was checked and finished using Canu v1.5 (https://github.com/marbl/canu/releases). If there was an overlap at both ends with 5,000 bp, then the sequence was looped and one end of the overlap was cut off. The final assembly generated a seamless chromosome with a depth of 1,170.69-fold coverage. Glimmer v3.02 (7), tRNA-scan-SE v2.0 (8), and barrnap v0.9 were used to predict protein-coding genes, tRNA, and rRNA, respectively (https://github.com/tseemann/barrnap/). CheckM v1.1.3 was used to check the quality of the assembled genome sequence with a completeness of 99.45%, contamination of 0%, and strain heterogeneity of 0% (9). Genome annotation was conducted using the NCBI Prokaryotic Genome Annotation Program (PGAP) v5.3 (10). Default parameters were used for all software unless otherwise specified.

The complete genome sequence contains a circular 2,041,530-bp chromosome with a GC content of 38.89%. Annotation yielded 2,076 protein-coding genes, of which 1,940 had functional assignments. Additionally, genes encoding 18 rRNAs, 69 tRNAs, and 4 noncoding RNAs were identified.

### Data availability.

The 16S rRNA gene sequence and complete genome sequence of the L. reuteri VHProbi M07 have been submitted to the GenBank database with the accession numbers ON965251 and CP089303, respectively. The raw reads were deposited at the Sequence Read Archive (SRA) with the accession numbers SRR17081379 and SRR20082667. The BioProject and BioSample accession numbers are PRJNA785010 and SAMN23525688, respectively.
